# Reduction of Soybean Meal Non-Starch Polysaccharides and α-Galactosides by Solid-State Fermentation Using Cellulolytic Bacteria Obtained from Different Environments

**DOI:** 10.1371/journal.pone.0044783

**Published:** 2012-09-11

**Authors:** Rafael Opazo, Felipe Ortúzar, Paola Navarrete, Romilio Espejo, Jaime Romero

**Affiliations:** Laboratorio de Biotecnología, Instituto de Nutrición y Tecnología de los Alimentos (INTA), Universidad de Chile, Santiago, Chile; Cairo University, Egypt

## Abstract

Soybean meal (SBM) is an important protein source in animal feed. However, the levels of SBM inclusion are restricted in some animal species by the presence of antinutritional factors (ANFs), including non-starch polysaccharides (NSPs) and α-galactosides (GOSs). The aim of this study was to reduce the soybean meal NSPs and GOSs by solid-state fermentation (SSF) using a combination of cellulolytic bacteria isolated from different environments (termites, earthworms, corn silage and bovine ruminal content). To analyse the key enzymatic activities, the isolates were grown in minimal media containing NSPs extracted from SBM. The selected bacterial strains belonged to the genera *Streptomyces*, *Cohnella* and *Cellulosimicrobium.* SSF resulted in a reduction of nearly 24% in the total NSPs, 83% of stachyose and 69% of raffinose and an increase in the protein content. These results suggest that cellulolytic bacteria-based SSF processing facilitates SBM nutritional improvement. In addition, the use of fermented SBM in animal diets can be recommended.

## Introduction

Defatted soybean meal is a global plant protein source for animal feedstuffs; however, its use is limited by the presence of several antinutritional factors (ANFs), including carbohydrates, such as α-galactosyl derivatives of sucrose (GOSs) and non-starch polysaccharides (NSPs) [1]–[3]. The principal soybean meal GOSs are stachyose and raffinose, with concentrations ranging from 2 to 5% w/w and 0.5 to 2% w/w of the dry matter (DM), respectively [1], [3]. The total soybean meal NSP concentrations are approximately 15–20% of the DM, and these NSPs are traditionally classified as cellulose, hemicellulose(s) and pectin(s) [3]–[5].

The antinutritional effects of GOSs and NSPs have been documented in different animal species. In swine, NSP and GOS supplementation adversely affects growth performance [1], [3], [6]. In poultry, NSPs disturb the digestibility of nutrients; the soluble portions of NSPs create viscous conditions in the small intestine, thus perturbing the activity of the digestive enzymes that are present. Furthermore, GOS depresses body weights, average daily gains and feed:gain ratios [1], [3]. In aquaculture species, NSPs and GOSs have also been considered ANFs [2]. In salmon diets, the presence of NSPs and GOSs alters the water and mineral content in faeces and obstructs the action of digestive enzymes [2]. The situation is critical in aquaculture because species such as salmon have higher protein requirements than poultry or swine [7]. The true challenge of the aquaculture industry is the replacement of fish meal with other protein ingredients [8], [9].

Alternative technologies have been proposed to reduce NSPs and GOSs. One such technology is soybean protein purification by alcohol/water extraction, which increases the protein content to nearly 65%. The use of soybean protein concentrates (SPC) versus SBM has been shown to improve the nitrogen and amino acid digestibility, feed intake, growth rate and feed efficiency ratio in animals [1], [10]. The benefits associated with the total or partial replacement of fish meal with SPC in salmon diets suggest that there is a significant role for SBM carbohydrates in antinutritional effects. However, the high cost of SPC has limited their incorporation into animal feed [8]. Another type of technology involves the incorporation of glycoside hydrolases into animal feed. However, in productivity assays, the effects of this alternative have been inconclusive in poultry, swine and salmon [1], [11], [12]. In salmon feed, the incorporation of cellulases, hemicellulases and pectinases presents an important restriction: the salmon body temperature range is between 10 and 12°C, but the optimal temperature for the activity of these enzymes is 50°C [11]. Another biotechnological method proposed for lignocellulolytic biodegradation in agricultural by-products is bacterial, yeast or fungal fermentation. In this area, solid-state fermentation (SSF) has proven to be a promising prospect [13].

In natural systems, NSPs are degraded principally by an enzymatic complex associated with cellulolytic bacterial and fungal consortia [14], [15]. GOSs are degraded by the enzyme α-galactosidase, which is produced by different microorganisms. Microorganisms with this enzymatic activity are expected to be found in different habitats, such as in soil [14], worms, insects, the superior animal gut [14], [16], [17], aquatic environments [14] and the phyllosphere [14].

Microbial fermentation has been used to reduce soybean meal ANFs, such as GOSs, glycinin and β-conglycinin [18]–[20]. The goal of this study was to isolate and select mesophilic, aerobic and cellulolytic bacteria and assess their ability to reduce soybean meal GOSs and NSPs by SSF.

## Materials and Methods

### Assessment of Environmental Processing, Bacterial Isolation and Cellulase Activity

The environments used to potentially isolate aerobic or facultative cellulolytic bacteria were as follows: garden soil sampled from a park, termites (*Neotermes chilensis*) obtained directly from an infested tree, earthworms (*Eisenia foetida*) obtained from a compost farm, bovine rumen content obtained from a slaughterhouse, rotten leaves sampled from a park and corn silage sampled from a dairy farm. The samples corresponded to different locations, which were each distant (kilometres) from the others. None of the sample collection sites were privately owned or protected in any way. Furthermore, the study did not involve endangered or protected species. The collected samples were transported to the lab and immediately processed. All substrates were ground, weighed and homogenised in sterile Phosphate-Buffered Saline (PBS buffer: 8.0 g NaCl, 0.2 g KCl, 1.44 g Na_2_HPO_4_•7H_2_O, 0.24 g KH_2_PO_4_, pH 7.4 in 1 L of water). To promote intestinal bacterial isolation, the earthworm sample was bathed superficially in 0.1% iodine for 5 min and washed 4 times in sterile PBS buffer. Serial dilutions of the homogenised substrates (10-, 100-, 1000- and 10000-fold) were plated onto solid minimal carboxymethylcellulose medium containing 5 g carboxymethylcellulose (Merck), 6 g NH_4_Cl, 0.6 g Na_2_HPO_4_, 15 g Bacto Agar, 10 mg Amphotericin B and essential trace elements, incorporated according to Hendriks (1995) [21] in 1 L of water. The medium was autoclaved at 121°C for 20 min. The plates were incubated for 5 days at 25°C, and the quantities of colonies obtained were expressed as CFU/g substrate. Subsequently, the cellulase activity was assessed in forty randomly selected strains from each environment by Congo Red staining [22]. The bacterial strains that manifested degradation halos were considered cellulolytic strains.

### Cellulolytic Strain Identification and Differentiation

Taxonomical identification of cellulolytic bacteria was performed by amplifying and sequencing the 16S rRNA gene. Genomic DNA was isolated from bacterial cultures using the Wizard Genomic DNA Purification Kit (Promega). The initial 16S rRNA amplification corresponded to positions 341 to 907 (*Escherichia coli* numbering); for a more stringent identification of the selected cellulolytic strains, the 16S rRNA genes were almost completely amplified from positions 27 to 1492 (*Escherichia coli* numbering). The PCR reaction was performed in a 30-µl reaction, containing 1.0 µl of genomic DNA from each strain and 29 µl of PCR reaction mix comprising 0.2 mM deoxynucleoside triphosphate (dNTPs), 1.5 U recombinant Taq DNA polymerase (Invitrogen), 2 mM MgCl_2_ and 0.25 pmoles/µl of each primer. The primers used were 341 and 907 for the short amplicon [23] and 27 and 1492 for the long amplicon [24] (Table 1). The PCR program involved an initial pre-denaturation of 3 min at 95°C, followed by 30 cycles of denaturation for 0.3 min at 95°C, primer annealing for 1 min at 58°C and extension for 1 min at 72°C and a final cycle of extension for 7 min at 72°C. The sequences were edited and matched to the Ribosomal Database Project (http://rdp.cme.msu.edu/) to identify the bacterial genera.

**Table 1:** Summary of primers used in the PCR amplification.

Target	Name	Sequence (given in 5′to 3′direction)	Reference
16S rDNA	341F	CCT ACG GGA GGC AGC AG	[23],[38]
16S rDNA	907R	CCG TCA ATT CMT TTG AGT TT	[23]
16S rDNA	788R	GGA CTA CCA GGG TAT CTA A	[37]
16S rDNA	27F	AGAGTTTGATCMTGGCTCAG	[24]
16S rDNA	1492R	GGTTACCTTGTTACGACTT	[24]
16S-23S spacer	L1F	GAA GTC GTA ACA AGG	[25]
16S-23S spacer	G1R	CAA GGC ATC CAC CGT	[25]

The intergenic transcribed spacers of the 16S-23S rRNA (ITS) were analysed to distinguish between the bacteria at the strain level. This analysis allowed us to distinguish between different and identical strains based on their ITS profiles. This analysis was helpful to focus the downstream enzymatic test. The ITS profile examination involved an initial analysis of 100 bacterial isolates. The PCR reaction was performed using the same protocol described above, but the ITS primers were L1 and G1 [25] (Table 1). The PCR conditions involved an initial pre-denaturation of 3 min at 95°C, then 30 cycles of denaturation for 3 min at 95°C, primer annealing for 1 min at 58°C and extension for 1 min at 72°C and a final extension for 7 min at 72°C. PCR products were visualised by polyacrylamide gel electrophoresis and silver staining, as previously described [25]. Differentiation of the ITS profiles was performed by binary data analyses, and the cluster distances were calculated using the DICE coefficient of similarity [26]. The dendrogram was generated using the unweighted pair-group average (UPGMA) hierarchical clustering method [27].

**Figure 1:**
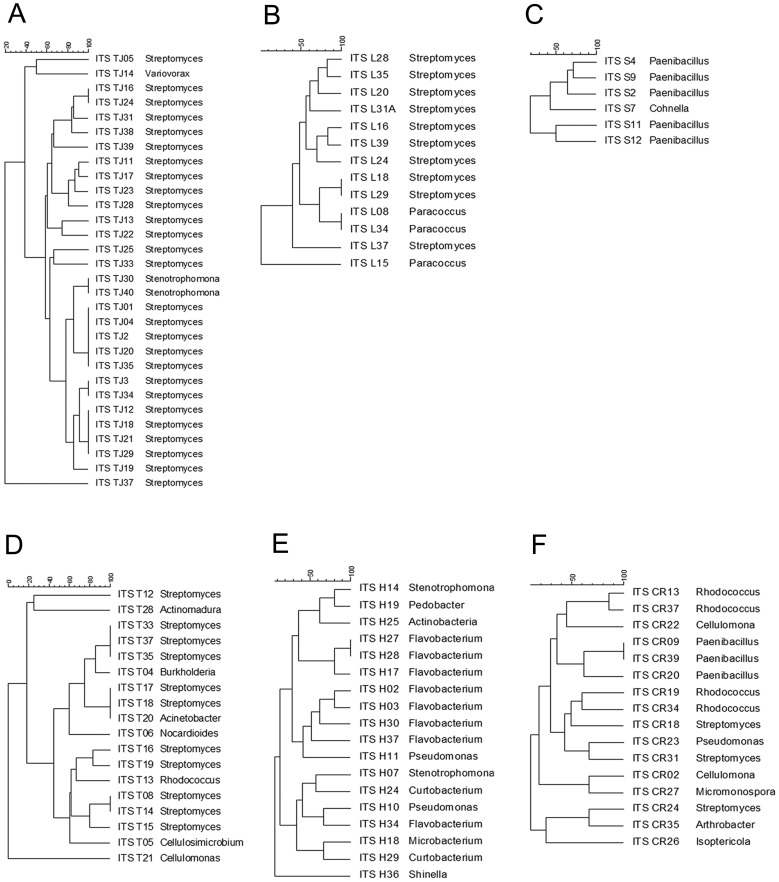
Dendrograms displaying ITS profile cluster analyses of the cellulolytic strains from each environment: (A) garden soil, (B) earthworm, (C) corn silage, (D) termite, (E) rotten leaves and (F) bovine rumen content. The profile distances between the cellulolytic strains were calculated based on the DICE similarity coefficient. Dendrograms were created using GelCompar II (Applied Maths) software with a position tolerance of 2%.

**Table 2:** Relative abundances of cellulolytic bacteria in the assessed environments.

Enviroments	Total CFU/gr^1^	Positive Congo red bacteria(n = 40)^2^	Relative Abundance	Number of Clusters^3^
Garden soil	1.8×10^7^	30	75%	20
Earthworm	3.7×10^7^	14	35%	11
Corn silage	6.0×10^3^	06	15%	6
Termites	2.9×10^6^	20	50%	13
BRC^4^	4.7×10^4^	20	50%	15
Rotted leaves	4.1×10^8^	23	57%	17
**Total**		113	47%	82

1 Total counts were assessed on minimal carboxymethylcellulose medium.

2 Congo Red-positive isolates were considered cellulolytic bacteria.

3 The number of different ITS profiles retrieved from each environment.

4 Bovine Ruminal Content.

**Figure 2:**
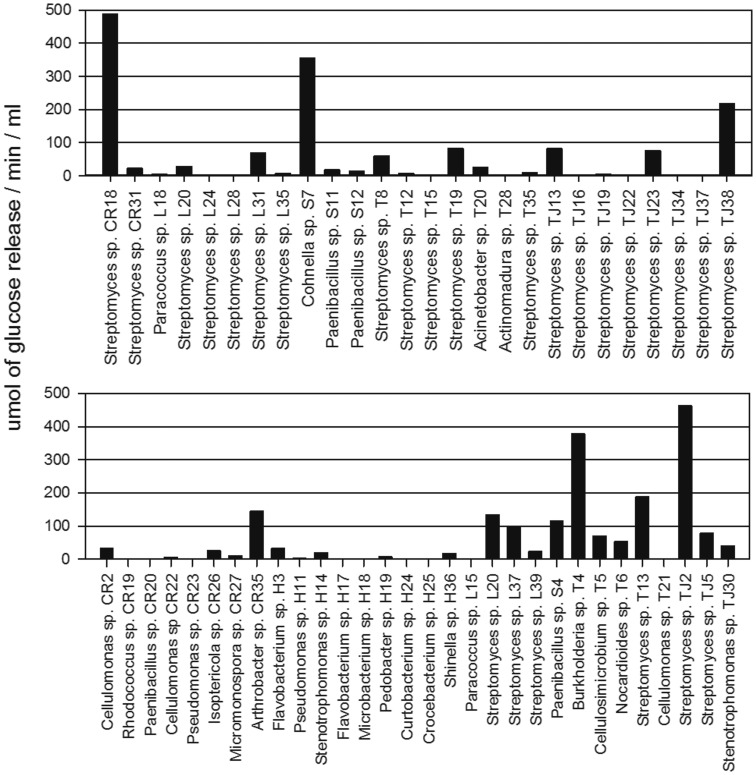
The total cellulase activity screen of the cellulolytic strains. The activity was assessed by filter paper hydrolysis. The strains were incubated beforehand in minimal medium containing NSP extracted from soybean meal. One unit of total cellulase activity was defined as the amount in µmoles of glucose released per min/ml of bacterial culture supernatant.

### Assessment of Enzymatic Activities

Three enzymatic activities were proposed for the selection of cellulolytic bacteria for SSF: the total cellulase (EC 3.2.1.4; EC 3.2.1.74; EC 3.2.1.91), endo-1,4-*β*-xylanase (EC 3.2.1.8) and 1,6-α-galactosidase (EC 3.2.1.22). The total cellulase and *β*-xylanase activities were tested first in a screening assay of the bacterial isolates. Subsequently, the selected strains were evaluated in a comparative assay, with eight replicates for each strain. The total cellulase and xylanase activities were assessed using supernatants collected from bacterial cultures in minimal medium containing NSP-rich SBM extract obtained from an intermediate step (after 85%–100% ethanol treatment, acetone wash and drying) according to the extraction protocol of Englyst et al. [28]. This SBM extract was free of monosaccharides, oligosaccharides and starch [28], and the NSP content was 30 mg per 100 mg of extract (laboratory determination). The enzymatic culture medium contained 20 mg of SBM Englyst extract, 12 mg NH_4_Cl and 1.2 mg Na_2_HPO_4_ in 2 ml of water. For each cellulolytic strain, 1×10^7^ cells were inoculated in 2 ml of liquid minimal SBM extract medium and incubated for 4 days at 25°C. The total cellulase activity was assessed using a modification of the filter paper method and an incubation period of 24 h at 50°C [29]. One unit of total cellulase activity was defined as the amount in µmoles of glucose released per minute per one ml of cultured bacterial supernatant [30]. The endo-β-xylanase and α-D-galactosidase activities were assessed using the p-nitrophenyl derivatives method; one unit of β-xylanase and α -galactosidase activity was defined as the µmoles of p-nitrophenol released per minute per millilitre of cultured bacterial supernatant at 25°C [31]. The α-D-galactosidase assay used a different minimal medium containing 20 g raffinose pentahydrate (Sigma, St. Louis, MO, USA), 6 g NH_4_Cl, 0.6 g Na_2_HPO_4_ and 2.5 g Bacto Yeast Extract in 1 L of water [32]. The criterion for strain selection was the presence of high enzymatic activity levels.

**Figure 3:**
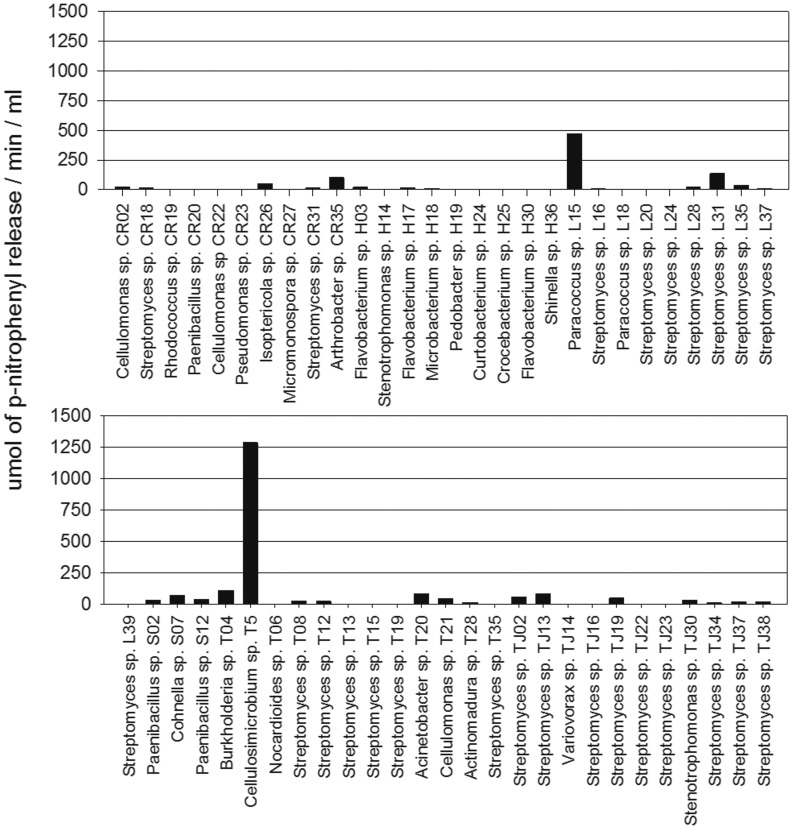
Endo-β-xylanase activity screening in cellulolytic strains. The activity was assessed using p-nitrophenyl-β-D-xylopyranoside as a substrate for hydrolysis. Bacteria were incubated beforehand in minimal medium containing NSP extracted from soybean meal; one unit of total xylanase activity was defined as the µmoles of p-nitrophenol released per min/ml of bacterial culture supernatant.

### Solid-state Fermentation

To estimate the effects of SSF on the reduction of soybean meal NSPs and GOSs, we organised two study groups. In the first group, each selected strain (inoculated group) was inoculated at 1×10^9^ cells/g of substrate; the second group was inoculated with culture buffer alone (non-inoculate group). Both groups involved 5 replicates each. Thirty grams of SBM was placed in glass flasks with filter aeration in a 2.4∶1 (v/w) weight ratio of water to substrate (wrw/s) with a relative humidity of nearly 85%. This was considered the substrate. SBM with a particle size of 400 µm was sterilised beforehand with 25 kilograys of gamma irradiation. The culture buffer was composed of 100 mM phosphate buffer, pH 7.0, containing 150 µg MgSO_4_•7 H_2_O, 36.5 µg CaCl_2_, 408 µg NH_4_Cl, 67 µg KCL, 30 µg FeSO_4_•7 H_2_O, 30 µg MnCl_2_•4 H_2_O and 30 µg ZnSO_4_•7 H_2_O per 30 g of substrate. The flasks were incubated with gentle shaking (5 rpm/min) for 10 days at 37°C [33].

**Table 3:** Taxonomic identification of cellulolytic strains selected for SSF.

Strain	Accessionnumber	Number of nucleotides	Taxonomic Affiliation	Closest relative Accession number	% identity
CR18	JF957187	1367	*Streptomyces pseudovenezuelae*	DQ462662	98%
S7	JF957188	1391	*Cohenella termotolerans*	EU867316	95%
T5	JF957189	1365	*Cellulosimicrobium sp.*	EU420065	99,8%

### Non-starch Polysaccharides, α-galactosides and Protein Quantification

The total levels of non-starch polysaccharides were quantified by spectrophotometric measurement [28]. Raffinose and stachyose were extracted as described by Giannoccaro et al. [34] and quantified by HPLC coupled to an Erma ERC-7510 Refractive Index Detector using a REZEX RSO oligosaccharide column (200 mm**⋅**10 mm; Phenomenex, Torrance, CA, USA) maintained at 60°C. HPLC-grade water was used for elution with a flow rate of 0.2 ml/min [35]. The protein levels were quantified by the Kjeldahl method [36].

### Dominant Strains after SSF

To assess the persistence and dominance of the inoculated strains at the end of the fermentation process, a specific restriction fragment length polymorphism (RFLP) assay was designed. This method allows for the identification of selected strains based on the specific and distinguishable fragments generated after digestion. Genomic DNA from the inoculated and non-inoculated fermentation vessels was isolated using the PowerSoil DNA isolation kit (Mo Bio). The 341 and 788 primers (Table 1) were used to amplify the 3 and 4 variable regions of the 16S rRNA gene [37], [38]. The amplicons were then digested by the restriction enzymes *Bmt I* and *BtsC I* (New England BioLabs). The RFLP reaction was performed using 1 U *Bmt I* and 2 U *BtsC I*, 1.0 µl of NEB buffer 2, and 1.0 µl amplified DNA in a 10-µl reaction volume. The digestion process consisted of a 3 h incubation at 50°C, then 2 h at 37°C and 1 h at 37°C with 1 µl of Proteinase K.

**Figure 4:**
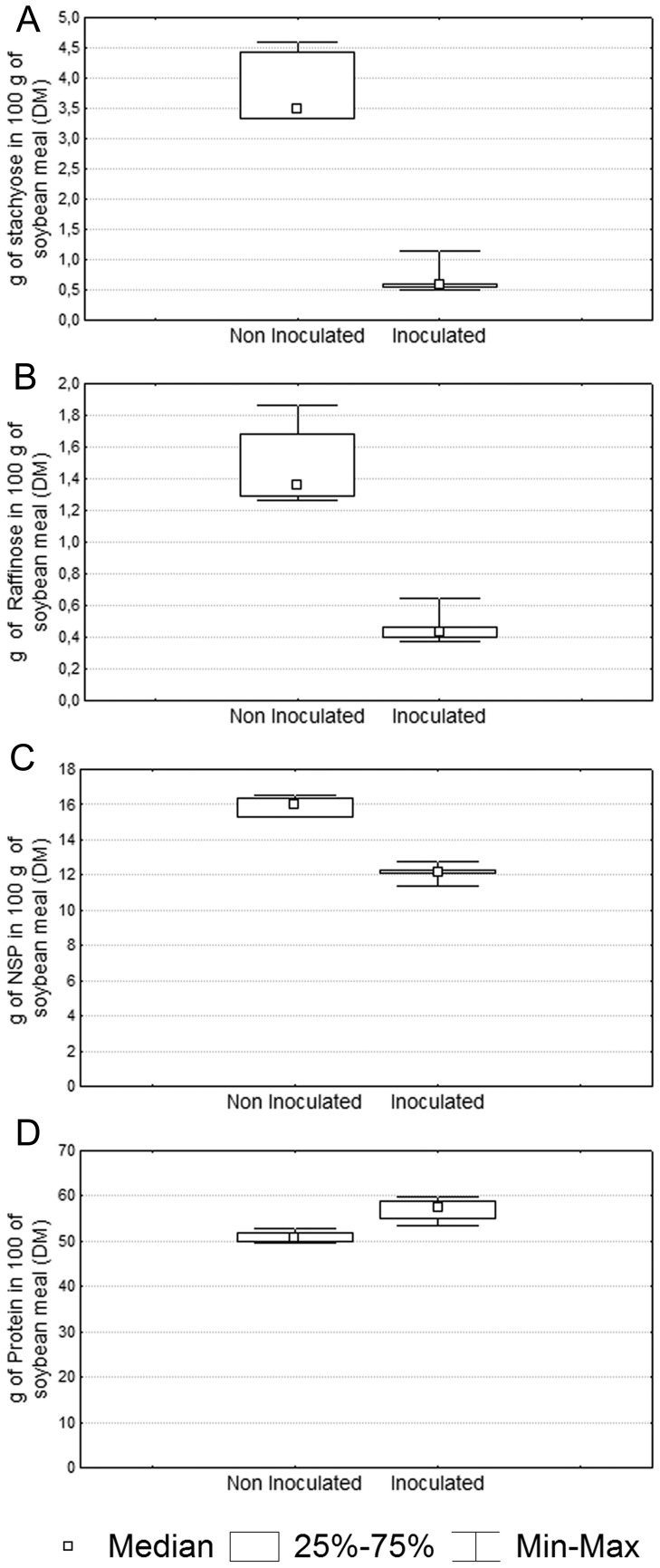
Box-and-whisker plots showing comparisons of the contents of (A) stachyose, (B) raffinose, (C) non-starch polysaccharide and (D) protein obtained after SSF between the inoculated group and the non-inoculated group. The results are expressed in grams of ANFs or protein per 100 g of FSBM.

### Statistical Analyses

The data were analysed using the Mann-Whitney U-test for comparing two groups, and non-parametric multiple comparisons of the mean ranks for all groups were used to test for multiple compared groups using Statistica 7.0 software [39].

**Figure 5:**
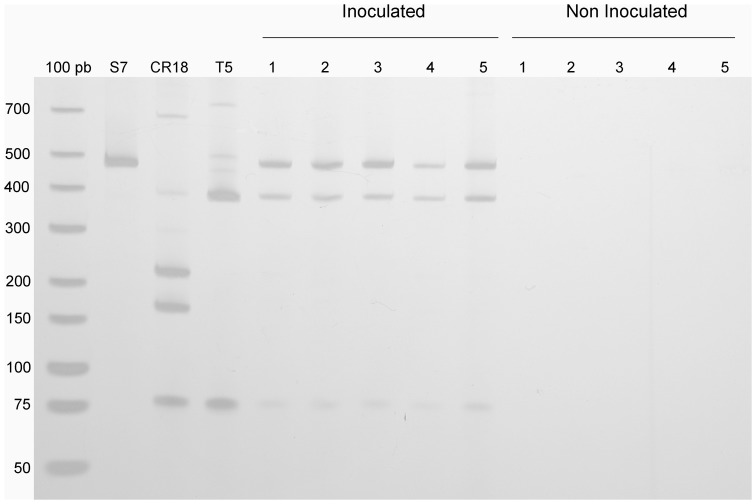
Dominance of the inoculated strains after SSF as revealed by their RFLP profiles. The RFLP profiles from the 16S rRNA genes of the selected strains (S7, CR18 and T5) were generated using a culture-independent approach. DNA was extracted directly from FSBM (inoculated and non-Inoculated groups), and the PCR amplicons were digested with the restriction enzymes *Bmt I* and *BtsC I.* The figure shows the RFLPs of the selected strains (S7, CR18 and T5) and the RFLP profiles from five replicates of inoculated and non-inoculated SSFs. The arrows indicate the presence of the S7 and T5 strains as dominant in the fermentation process.

## Results

### Identification of Isolates Retrieved from Each Environment

Of the 240 colonies that were randomly collected from the six environments, 113 isolates were selected based on the presence of degradation halos in carboxymethylcellulose medium after Congo Red staining. However, some strains were discarded because they exhibited no growth in subcultures after the Congo Red analyses. From the viable strains, the 16S rRNA sequencing and comparison with databases identified 23 bacterial genera. For further characterisation, the spacer region (ITS) between the 16S and 23S rRNA genes was analysed. Based on their ITS profiles, 82 unique strains were identified. Simultaneously, cluster analyses were performed for each environment (Figure 1). Table 2 summarises the data from each environment, including the total cultivable bacterial counts, relative abundances of cellulolytic bacteria and numbers of ITS profiles.

### Abundance and Diversity of Cellulolytic Strains from the Six Different Environments

Four bacterial phyla were represented by the isolated cellulolytic strains. *Actinobacteria* was the most abundant group (52%), followed by *Proteobacteria* (30%), *Firmicutes* (9%) and *Bacteroidetes* (9%). At the genus level, *Streptomyces* was the most abundant genus, isolated from four of the six environments with a relative abundance of 18%–90%. From the comparison of the genera abundance between the environments, the garden soil, earthworms and corn silage exhibited limited genus diversity (2 or 3 bacterial genera). In contrast, the termites, bovine ruminal content and rotten leaves contained more than 7 bacterial genera (Figure 1). The garden soil contained the highest abundance of cultivable cellulolytic bacteria (75%). Ninety per cent of these cellulolytic strains belonged to the genus *Streptomyces,* and 60% had unique ITS profiles. Similarly, 70% of the cellulolytic strains identified from earthworms belonged to the genus *Streptomyces,* and 90% showed distinctive ITS profiles. The cellulolytic bacteria identified from termites belonged to seven different genera, mainly *Streptomyces* (61%); the other genera were represented by only one strain each with unique ITS profiles (e.g., *Cellulosimicrobium* or *Cellulomonas*) (Figure 1).

The most common cellulolytic bacteria in the bovine ruminal content was *Rhodococcus* (25%), followed by *Streptomyces* (19%); all of the strains had unique ITS profiles. The corn silage contained the lowest relative abundance of cellulolytic bacteria; all of identified strains belonged to the *Paenibacillus* or *Cohnella* genera, and each strain had unique ITS profiles (Figure 1). The most common genus in rotten leaves was *Flavobacterium* (40%), and the other genera were represented by only one or two strains (Figure 1).

### Screening Key Enzymatic Properties in Cellulolytic Bacterial Isolates

Three enzyme activities (cellulase, β-xylanase and α-galactosidase) were considered critical requisites for definitive bacterial selection based on the structures of the NSP and GOS glycosidic linkages. The NSPs present a more complex structure than the GOSs because they are a mixture of polymers, namely, cellulose, hemicellulose and pectins. Among these polymers, cellulose is a homogeneous polysaccharide composed of D-glucose subunits linked by β-(1–4) glycosidic bonds, which are degraded by cellulase systems that specifically hydrolyse these bonds [40]. Because cellulose comprises 30% (w/w) of the NSPs in soybean meal [5], this homopolymer was the first target for NSP degradation using SSF. In the total cellulase screen, four strains showed high enzymatic activity (150 µmol of glucose released/min/ml): S7 (*Cohnella*), CR18 (*Streptomyces*), TJ38 (*Streptomyces*) and T4 (*Burkholderia*) (Figure 2). In comparative assays, S7 and CR18 exhibited the highest total cellulase activities, exhibiting significant differences from the other preselected bacteria (p<0.05).

In contrast, hemicellulose and pectins are heterogeneous polysaccharides that require several different glycoside hydrolase enzymes for their degradation. Therefore, endo-1,4-*β*-xylanase, the enzyme that hydrolyses the xylose backbone present in hemicellulose, might be a useful complementary activity for NSP degradation [40]. Only two strains exhibited high β-xylanase activity (more than 400 µmol of p-nitrophenyl release/min/ml): T5 (*Cellulosimicrobium sp*.) and L15 (*Paracoccus sp*.), Figure 3. In the β-xylanase comparative assay, no significant differences (p<0.05) between these strains were observed.

The GOSs in soybean meal are oligosaccharides that predominantly consist of one or two 1,6-α linked units of galactose attached through 1,3-α bonds to a terminal sucrose [3]. Sucrose disaccharide hydrolysis occurs in the digestive tract of animals by the enzyme saccharase. Therefore, GOS reduction is focused in the hydrolysis of the 1,6-α-galactose bonds by α-galactosidase. This enzymatic activity was assessed in the selected strains and was observed in the S7 and CR18 strains.

Thus, the strains selected for the SSF process were S7 (*Cohnella*) and CR18 (*Streptomyces*), both of which exhibited high total cellulase and α-galactosidase activities, and strain T5 (*Cellulosimicrobium*), which showed endo-1,4-*β*-xylanase activity. For improved identification of the selected strains, the 16S rRNA genes were sequenced almost completely; the results are summarised in Table 3.

### Solid-state Fermentation Results

The reduction in the soybean meal GOSs and NSPs was determined by comparing the GOS and NSP contents in cellulolytic bacteria-fermented SBM (FSBM), known as the inoculated group, and the SBM processed under the same conditions but without bacteria, known as the non-inoculated group. The median stachyose and raffinose levels in the inoculated group were lower than those in the non-inoculated group, 0.44 g vs. 3.52 g and 0.48 g vs. 2.11 g per 100 g soybean meal (DM), respectively (Figure 4). These ANFs were reduced by 88% for stachyose and 76% for raffinose, and these differences were statistically significant (p<0.05). The inoculated group also exhibited 23.6% lower NSP levels than the non-inoculated group, with medians of 12.17 g vs. 16.01 g per 100 g of soybean meal (DM); this difference was statistically significant (p<0.05), Figure 4C. Another very important issue was the enrichment of protein content in the FSBM. The inoculated group manifested higher protein levels (57%) than the non-inoculated group (51%). This difference was statistically significant (p<0.05) Figure 4D.

The selected cellulolytic strains CR18 (*Streptomyces*), S7 (*Cohnella*) and T5 (*Cellulosimicrobium*) could be distinguished by their 16S rRNA gene sequences. Therefore, the presence of these strains can be detected by culture-independent analysis using restriction enzyme digestion of the 16S PCR amplicons (specific RFLP profiling). The dominance of the selected cellulolytic strains was assessed at the end of the fermentation process by RFLP profile analysis. As shown in Figure 5, only the T5- and S7-specific RFLP profiles were detected.

## Discussion

Our results show that SSF using a combination of the selected cellulolytic bacteria significantly reduced the NSP and GOS levels and enriched the protein contents. Therefore, this SSF is a promising method that could lead to the increased incorporation of SBM into animal or aquaculture diets. This process was developed based on the selection strategy of environmental cellulolytic bacteria. This novel strategy consisted of the selection of bacterial strains for soybean NSP degradation based on bacterial incubation in a minimal medium formulated with a soybean NSP-rich extract (obtained using the Englyst method). This medium mimicked the soybean meal environment, including the presence of NSP without simple sugars, leading to the identification of strains exhibiting enzymatic activities appropriate for NSP degradation.

Candidate bacterial strains for the process were isolated from the different environments because it is well known that these environments harbour cellulolytic bacteria [14], [16], [17], [41]. However, the scarcity of available numerical data related to the relative abundance of cellulolytic bacteria in different environments allows only a limited comparison of our results. From the termite *Neotermes chilensis,* the relative abundance of cellulolytic bacteria was inferior to that obtained from the termite *Zootermopsis angusticollis* (1×10^7^ CFU/ml), although this difference may be explained by the different methods used to assess cellulolytic activity [42]. In contrast, the rotten leaves and garden soil exhibited superior relative abundances compared to horticultural waste (1×10^5^ CFU/g) [43] or different soil samples (1×10^5^ CFU/g) [21]. The relative abundance of cellulolytic bacteria in the bovine ruminal contents was not previously known, as the most abundant cellulolytic bacteria in this environment are anaerobic, and previous studies have focused on these anaerobic species [17].

Among the identified cellulolytic bacteria, most of the bacterial isolates belonged to the phylum *Actinobacteria* (52%), mainly represented by the genus *Streptomyces*. The *Actinobacteria* phylum has been associated with multiple enzymatic activities and bioactive substances, including cellulase, pectinase and hemicellulase as well as heterotrophic nitrification and the excretion of various antibiotics or bacteriocins [44]–[46]. Aerobic *Actinobacteria* utilise systems comprising independently functioning enzymes, often with carbohydrate binding domains, in contrast to widely described cellulolytic anaerobes, including *Clostridium* species, which organise cellulases and other glycosyl hydrolases into large complexes known as cellulosomes [47]. The genome analysis in *Streptomyces* suggested that this bacterial group might have evolved by frequent gene duplication or lateral gene transfer events, which play a role in shaping the genome and in the acquisition of novel gene functions to adapt to the extremely variable soil environment, where this group acts as the decomposers of organic substances for carbon recycling [48]. These capabilities may explain the dominance of this group in some of the environmental samples and suggest that *Streptomyces* may be an interesting alternative to include in the bacterial combination used to reduce the soybean meal ANFs.

Additional examples of bacterial isolates taxonomically related to the cellulolytic strains selected in this study have been proposed for agro-industrial residue degradation. For example, *Streptomyces* strains have been previously used in SSF focused on lignocellulolytic degradation in sugarcane bagasse [13]. *Bacillus* taxonomically related to *Cohnella* have also been proposed for lignocellulolytic degradation in rice straw [13] and for protein enrichment of corn cob heteroxylan [49]. Members of the *Cellulosimicrobium* genus have been documented for their xylanase activities [50].

This study yielded satisfactory ANF degradation levels when the soybean meal was fermented with a combination of cellulolytic bacteria. Soybean fermentation is an ancient process developed in Asian cultures approximately 1500 years ago [51], where it was likely used as a natural process to preserve soy food or for incorporating other tastes into soy food. In addition, previous studies have aimed to reduce the soybean meal ANFs by controlled bioprocesses using microorganisms [19], [20]. One of these studies has proposed fermentation with *Lactobacillus brevis*, which yielded 10.2% GOS degradation [19]. The other used *Debaryomyces hansenii* UFV-1 and showed a 100% GOSs degradation [20]. Both fermentation studies were performed using high weight ratios of water to substrate (wrw/s), 3.5∶1 (v/w) and 10∶1 (v/w), respectively. This point is important because the resulting fermented meal must be dried to control future fungal contamination, increasing the cost of fermented soybean meal (FSBM). In this study, preliminary SSF assays indicated that the optimum wrw/s was 2.4∶1 (v/w). Any reduction in the water ratio diminished the NSP degradation after the process. Temperature is another important factor for NSP degradation, which has been previously documented [52]–[55]. In this study, preliminary SSF assays using temperatures of 20–25°C indicated that at these temperatures, no reduction in GOS or NSP occurred; ANF degradation required higher temperatures, such as 35°C. This tendency is consistent with the temperature of 30°C used by Rodrigues Brasil et al. [20] that was required to degrade 100% of GOS. However, Refstie et al. (2005) [19], did not achieve significant levels of GOS degradation at this temperature. Furthermore, a swine nutritional study using FSBM has described a reduction in the levels of glycinin and β-conglycinin; however, the author did not describe the fermentation process [18].

An increase in the protein content following SSF has been previously documented for agro-industrial residues [55]–[58]. This increase in protein levels may be explained by a reduction in the amount of carbohydrates due to bacterial carbohydrate metabolism and respiration, which can lead to carbon mineralisation (CO_2_). Other sources of protein to be considered include the bacterial inoculum and ammonia from the buffer. The contribution of ammonia was negligible because of the small amounts in the media and the presence of proteins and amino acids from the SBM. The inoculum protein contribution was estimated to be 30 mg/g of SBM (DM) based on the total number of bacteria and the protein contents of each bacterial cell (300 fg) [59]. This corresponds to a small proportion compared to the protein increase observed in FSBM (DM), estimated at 600 mg/g. The enrichment in the protein content after fermentation is a very appealing benefit to boost the inclusion of soybean meal in aquaculture diets because the increased protein content after FSBM means that less FSBM is required compared to soybean meal (SBM) to achieve the protein requirements of an animal diet formulation. Thus, diets formulated with FSBM will contain less ANFs than diets containing SBM. Thus, the diets formulated with FSBM incorporate less soybean meal ANFs for two reasons: the first is due to the reduction in indigestible carbohydrates (GOS and NSPs) and the second is due to the enrichment in protein content that reduces soybean meal ANFs in the diets.

In conclusion, the benefits of FSBM by SSF using a cellulolytic bacteria consortium is an appealing option for reducing the soybean meal ANFs and increasing the use of soybean meal in animal or aquaculture diets. However, it will be necessary to optimise SSF for large-scale production, evaluate the quality and digestibility of FSBM proteins and perform *in vivo* assays to evaluate the nutritional benefits of the inclusion of FSBM in animal or aquaculture diets.
